# Efficacy of Liraglutide for Weight Loss in Overweight and Obese Non-diabetic Adults: A Systematic Review and Meta-Analysis of Randomized Controlled Trials

**DOI:** 10.7759/cureus.82479

**Published:** 2025-04-17

**Authors:** Néstor Israel Quinapanta Castro, Mishell Almeida, Andres F Orbea, Domenica N Andrade, Jonathan Flores Carrera, Francisco Yepez Vargas, Mariela L Carrasco

**Affiliations:** 1 Department of Research, Regional Autonomous University of the Andes (UNIANDES), Ambato, ECU; 2 Department of Medicine, Central University of Ecuador, Quito, ECU; 3 Department of Medicine, Regional Autonomous University of the Andes (UNIANDES), Ambato, ECU; 4 Department of Occupational Health, Pontifical Catholic University of Ecuador, Quito, ECU; 5 Department of Emergency Medicine, Ambato General Hospital (IESS), Ambato, ECU

**Keywords:** liraglutide, non-diabetic adults, obesity and overweight, placebo controlled trials, weight loss and obesity

## Abstract

Liraglutide is commonly used in the management of type 2 diabetes and obesity and has been part of clinical practice for several years. However, evidence on its efficacy and safety in people without diabetes remains limited. The aim of this review is to analyse the efficacy of liraglutide in non-diabetic obese or overweight adults by systematically reviewing and meta-analyzing clinical trials.

A systematic peer review was performed according to the Preferred Reporting Items for Systematic Reviews and Meta-Analyses (PRISMA) protocol using PubMed/MEDLINE, Scopus, and Web of Science databases. Meta-analysis was performed using a random effects model in Review Manager 5.7.0 (The Cochrane Collaboration, Oxford, UK). The quality of the evidence was evaluated using the Grading of Recommendations Assessment, Development, and Evaluation (GRADE) approach. Risk of bias was assessed using the Cochrane Risk of Bias 2.0 (RoB 2.0) tool for randomized trials. Publication bias was analyzed using funnel plots. It was registered in PROSPERO (International Prospective Register of Systematic Reviews) under the number CRD42025637238.

Eleven randomized clinical trials were included for analysis in this study. Of 92 studies assessed for eligibility, 11 trials were included, with a combined total of 1328 patients; the mean (SD) age was 44.49 (7.04) years, with a proportion of males (21.36%, n = 319) and females (78.77%, n = 1009), respectively. Compared with control groups, a more significant weight loss was seen in liraglutide groups with an overall mean difference of -4.59 kg, 95% confidence interval (CI) -6.02 to -3.15 (I^2 ^= 86%). The overall analysis results showed that liraglutide improved weight control. There were also significant reductions in waist circumference (-3.22 cm, 95% CI -3.77 to -2.67), BMI (-1.71 kg/m^2^*,* 95% CI -2.45 to -0.96), but not in HbA1c (-0.43%, 95% CI -1.13 to 0.27).

Liraglutide effectively reduces body weight, percentage weight loss, waist circumference, and BMI, making it a therapeutic option for the management of non-diabetic patients with obesity and overweight.

## Introduction and background

The rapid global increase in obesity, a phenomenon known as "globesity," is one of the most important public health challenges of our time [1]. In 1997, the World Health Organization (WHO) declared it a global epidemic [1,2]. According to recent WHO estimates [3], more than 2.5 billion adults over 18 years of age are overweight, and more than 890 million are living with obesity. The age-adjusted prevalence of obesity increased from 4.6% in 1980 to 14.0% in 2019 [4]. This condition is associated with an increased risk of all-cause mortality ranging from 21% to 108% [5]. It also significantly increases the risk of major cardiovascular events, such as acute myocardial infarction (AMI) and stroke, particularly in individuals with a body mass index (BMI) ≥35 kg/m^2^ (hazard ratio (HR): 4.71) [6].

Dipeptidyl peptidase-4 (DPP-4) inhibitors and glucagon-like peptide-1 (GLP-1) receptor agonists (RAs) are important treatments for type 2 diabetes [7]. The former are taken orally and the latter are administered subcutaneously. Both lower blood glucose with a low risk of hypoglycemia, but GLP-1 RAs show greater efficacy, aid in weight loss, and improve cardiovascular factors [7]. The GLP-1 RA liraglutide is an innovative therapeutic option for glycaemic control with additional benefits in reducing body weight and the risk of cardiovascular events [8]. This incretin analogue antidiabetic agent acts on pancreatic β-cells to stimulate endogenous insulin secretion, contributing to glycaemic control and appetite suppression [8,9].

Liraglutide was approved in 2010 for the treatment of type 2 diabetes mellitus [9]. Its use in obesity is recommended at a dose of 0.6 to 3.0 mg/day [8,10,11]. However, evidence on its effectiveness and safety in people without diabetes remains limited [12].

In this context, a review of studies with high methodological quality in non-diabetic subjects will allow a more accurate assessment of the effect of liraglutide on weight loss and its short- and long-term outcomes. This may contribute to the identification of new therapeutic strategies for the management of overweight and obesity in this population. Therefore, the aim of this review is to analyse the efficacy of liraglutide in non-diabetic obese or overweight adults by systematically reviewing and meta-analyzing clinical trials.

## Review

Methods

The systematic review was conducted according to the Preferred Reporting Items for Systematic Reviews and Meta-Analyses (PRISMA) guidelines, which provide a set of standards to ensure transparency and quality in the conduct and reporting of systematic reviews. The research question was formulated using the PICO (Patient, Intervention, Comparison, Outcome) framework, a tool designed to clearly structure the key components of the question, thereby facilitating the identification and selection of relevant studies.

A comprehensive search was performed using a limited vocabulary based on medical subject headings (MeSH) terms and free text keywords. The strategy considered spelling variations, synonyms, acronyms, and truncations to identify relevant studies. Field labels and Boolean operators were used, with language restrictions to English and Spanish. The search strategy included terms such as "liraglutide" combined with "obesity," "overweight," "weight loss," and "adults" and "young adults. Electronic databases such as PubMed, MEDLINE, Scopus, and Web of Science were searched for records from 2019 to February 2025. In addition, reference lists of selected studies were reviewed.

This review included randomized, placebo-controlled clinical trials. The intervention studied was liraglutide 3 mg/day administered by subcutaneous injection. The study population consisted of adults (≥18 years) of any sex or ethnicity with a BMI >25 kg/m^2^ and without type 1 or type 2 diabetes, gestational diabetes, or psychiatric disorders.

Studies conducted outside of this period, letters to the editor, animal studies, and articles without full-text access were excluded. In addition, to ensure that the results accurately reflect the target population, individuals using liraglutide as maintenance therapy after surgical or pharmacological interventions and studies involving only prediabetic individuals were excluded.

Two independent investigators (I.Q., L.C.) selected studies from different databases using predefined search strategies and eligibility criteria. In case of disagreement about inclusion or exclusion, a third investigator (F.Y.) was consulted to resolve the issue by mutual consensus.

Data extraction was performed using a form based on the Cochrane Consumers and Communication Group extraction template, which was adapted to assess the efficacy and safety of liraglutide for weight reduction in adults with obesity or overweight without diabetes, according to published clinical trials. The extracted data included authors, database consulted, journal of publication, publication date, study location, article type, DOI, original title, full abstracts, methodology used, and results obtained. The extraction process was conducted independently by two reviewers (N.Q., M.A.), with discrepancies resolved by discussion with a third investigator (F.Y.) when necessary. Missing data were resolved by contacting the authors of the studies when necessary.

The primary outcomes included weight loss (kg), percentage change in body weight, and waist circumference (cm). Secondary outcomes included changes in BMI (kg/m^2^) and glycated hemoglobin (HbA1c%).

Treatment effect measures included mean difference (MD) or standardized mean difference (SMD) for continuous data. Cochrane Review Manager 5.7.0 (The Cochrane Collaboration, Oxford, UK) was used for statistical analysis and synthesis of the extracted data. A 95% confidence interval (95% CI) and a p-value of 0.05 were used. Statistical heterogeneity was assessed using the Chi^2^ test and the I^2^ statistic, with a threshold of 50% indicating substantial heterogeneity, in which case a random effects model was applied. In addition, publication bias was assessed using funnel plots and Egger's test when at least 10 randomized controlled trials (RCTs) were included.

The quality of evidence was assessed using the Grading of Recommendations Assessment, Development, and Evaluation (GRADE) approach. GRADE takes into account risk of bias, inconsistency, indirectness, imprecision, and publication bias. The potential for bias in the included studies was assessed by two independent authors (N.Q., M.A.) using the Cochrane Risk of Bias (RoB 2.0) tool. The overall risk of bias was assessed according to the criteria of Tramacere (2015), including low, high, and uncertain risk categories. Disagreements were resolved by consensus or expert opinion.

This systematic review is registered in PROSPERO (International Prospective Register of Systematic Reviews) under the number CRD42025637238.

Results

Search Results

**Figure 1:**
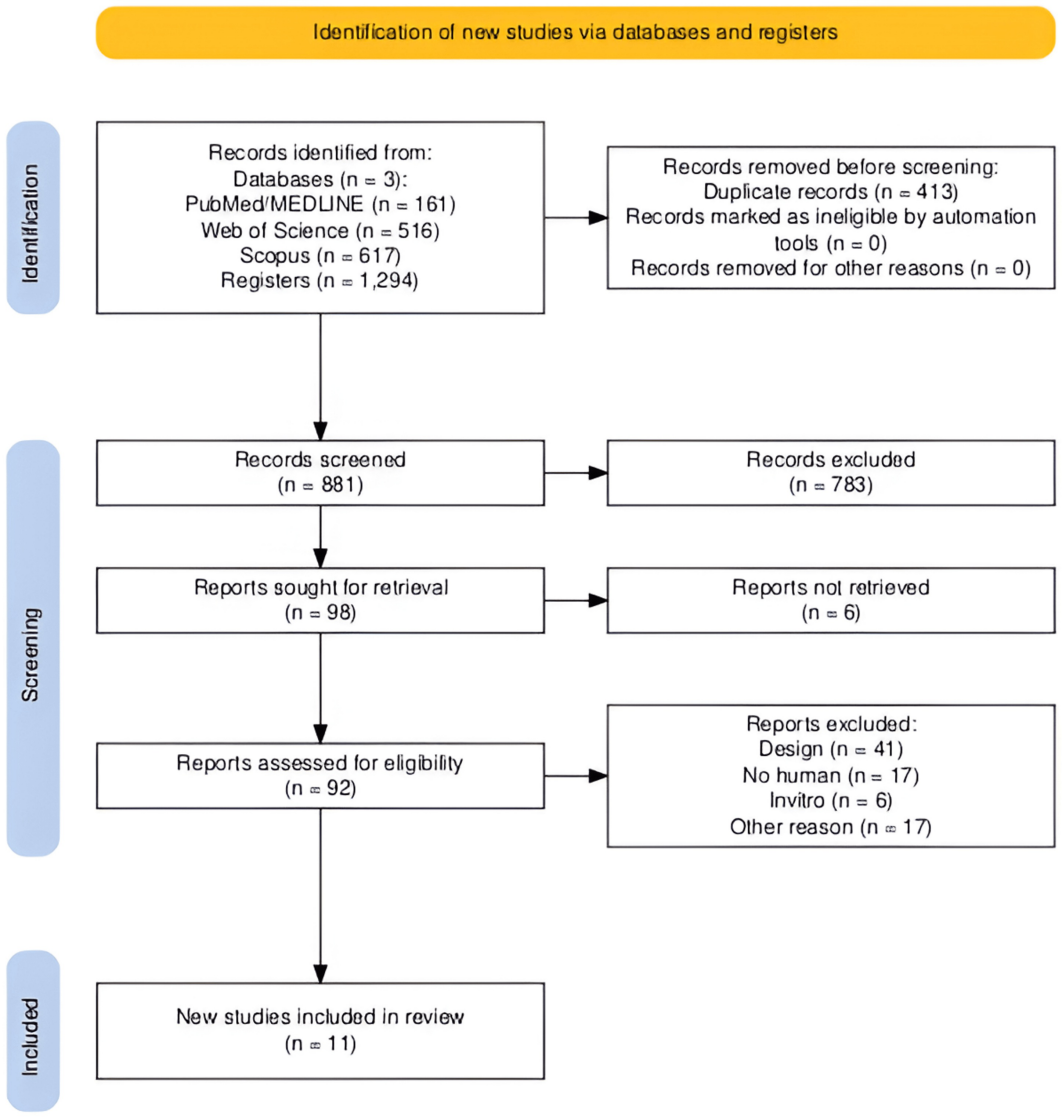
PRISMA flow diagram of the study selection process PRISMA: Preferred Reporting Items for Systematic Reviews and Meta-Analyses

In the present review, 1294 references were identified from the electronic search of databases. After elimination of duplicates (n = 413) and initial screening by title and abstract, 783 references were excluded, and 98 references were eligible for full-text analysis. After applying the inclusion and exclusion criteria, 41 studies were excluded due to incompatible study design, 17 because they were non-human studies, six for being in vitro research, and finally 17 for other reasons. Therefore, 11 studies were included for analysis in this study, as shown in the PRISMA flowchart (Figure 1).

Characteristics of Included Studies

Table 1 shows the characteristics of the included studies. With a total of 1328 patients, the mean (SD) age was 44.49 (7.04) years, with a proportion of males (21.36%, n = 319) and females (78.77%, n = 1009), respectively. The mean (SD) BMI was 37.38 (2.93) kg/m^2^. The studies were conducted in three regions: eight studies in the United States, two in Denmark, and one in Switzerland.

**Table 1:** Baseline characteristics of the included studies BMI: Boby mass index; RCCT: Randomized controlled clinical trial; IBT: Behavioral intervention; JNJ-64565111: Dual agonist of glucagon-like peptide-1 and glucagon receptors; * 3.0 mg/day of liraglutide subcutaneously

Reference	Year	Country	Sample size	Liraglutide 3.0 mg	Placebo	Design	Weeks of intervention	Arms	Mean age (SD/95% CI)	Mean BMI (kg/m^2^) (SD/95% CI)
Rubino et al. [13]	2022	USA	n=212	n=127	n=85	Phase 3 RCCT	68	Semaglutide 2.4 mg/semana vs. liraglutide* vs. placebo	49	37.5
Maselli et al. [14]	2022	USA	n=136	n=67	n=69	RCCT	16	Liraglutide* vs. placebo	39.6 (30.65 to 48.1)	35.75 (32.85 to 39.95)
Wadden et al. [15]	2020	USA	n=282	n=142	n=140	Phase 3b RCCT	56	Liraglutide* + IBT vs. placebo + IBT	47.2 (11.4)	39.0 (7.0)
Coppin et al. [16]	2023	Switzerland	n=44	n=20	n=24	RCCT	16	Liraglutide* + counseling vs. placebo + counseling	38.22 (12.7)	35.39 (2.94)
Alba et al. [17]	2021	USA	n=179	n=119	n=60	Phase 2 RCCT	26	JNJ-64565111 5 mg vs. JNJ-64565111 7.4 mg vs. JNJ-64565111 10 mg vs. liraglutide* vs. placebo	46.3 (11.7)	40.5 (4.1)
Tronieri et al. [18]	2019	USA	n=73	n=37	n=36	RCCT	52	IBT vs. IBT+ liraglutide* vs. IBT+ liraglutide* + diet	46.7 (12.2)	38.8 (4.8)
Elkind-Hirsch et al. [19]	2022	USA	n=67	n=44	n=23	RCCT	32	Liraglutide* vs. placebo	31.3 (0.9)	42.75 (1.43)
Saxena et al. [20]	2021	USA	n=54	n=28	n=26	RCCT	6	Liraglutide* vs. placebo	45.4 (11.9)	34.5 (2.8)
Allison et al. [21]	2022	USA	n=27	n=13	n=14	RCCT	17	Liraglutide* vs. placebo	44.5 (10.5)	37.9 (11.8)
Lundgren et al. [22]	2021	Denmark	n=98	n=49	n=49	RCCT	52	Liraglutide* vs. placebo vs. exercise vs. exercise + liraglutide*	42 (12)	37 (2.9)
Gudbergsen et al. [23]	2023	Denmark	n=156	n=80	n=76	RCCT	52	Liraglutide* vs. placebo	59.2 (10.3)	32.1 (4.9)

Primary and Secondary Outcomes

**Figure 2:**
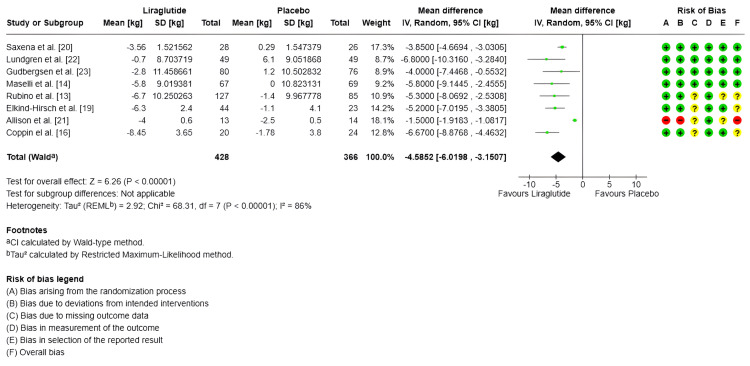
Forest plot and risk of bias: change in body weight (kilograms) IV: Inverse variance; Random: Random-effects model; Std.: Standardized; 95% CI: 95% confidence interval; + Low risk of bias; ? Some concerns; - High risk of bias References: [20,22,23,14,13,19,21,16]

Body weight (kg): Only eight studies measured body weight in kg, with a total of 428 subjects in the experimental group and 366 in the control group [13,14,16,19-23]. Regarding the combined effect, for the random-effects model, the MDs evidenced a significant impact in the decreased of body weight of -4.59 kg (95% CI -6.02 to -3.15) (SMD -1.26 95% CI -1.86 to -066) compared to the control group (p < 0.00001); however, there is high heterogeneity between studies (I^2^ = 86%) (Figure 2).

**Figure 3:**
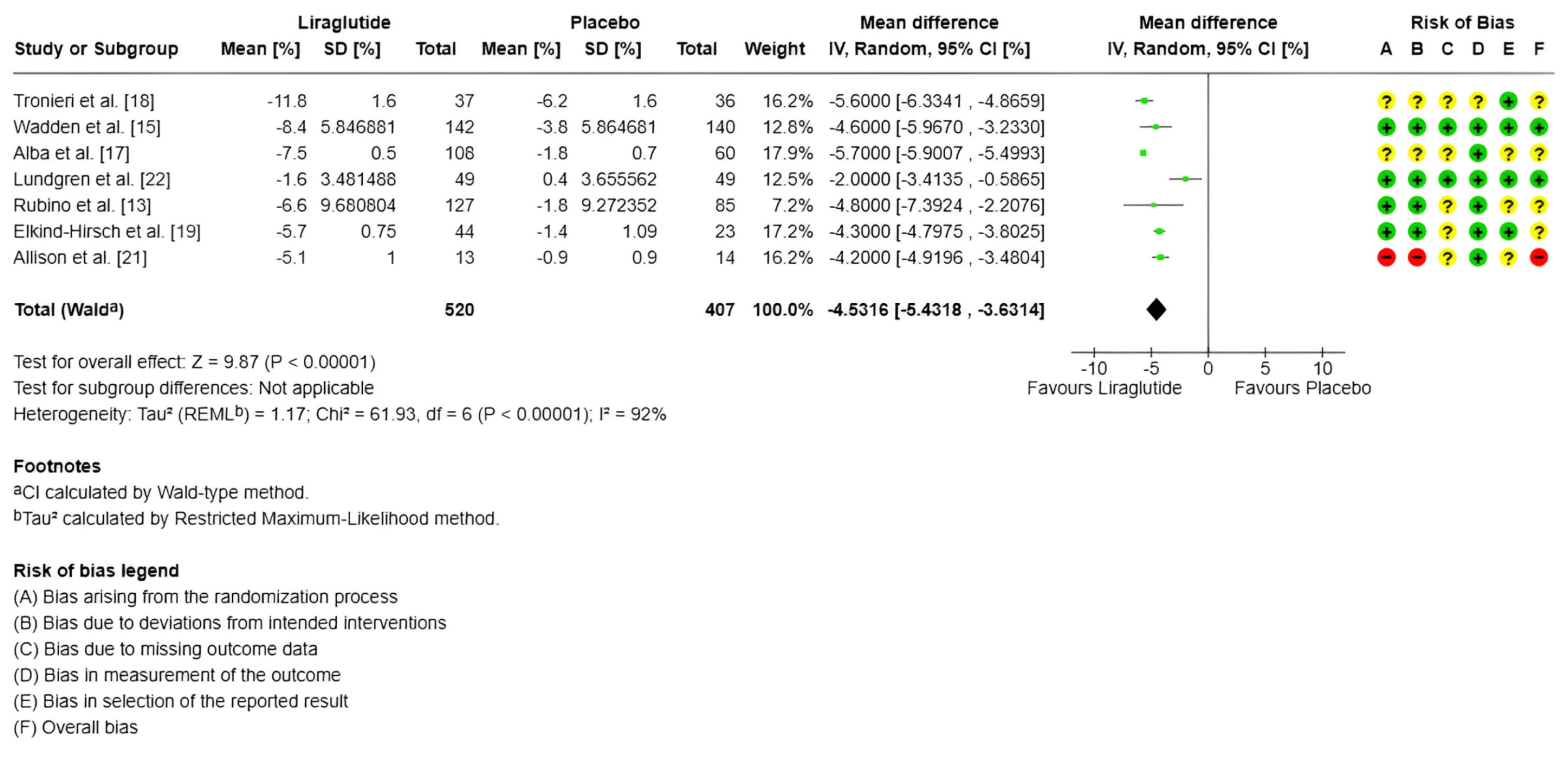
Forest plot and risk of bias: percentage of body weight loss IV: Inverse variance; Random: Random-effects model; Std.: Standardized; 95% CI: 95% confidence interval; + Low risk of bias; ? Some concerns; - High risk of bias References: [18,15,17,22,13,19,21]

Body weight (%): Only seven trials measured the percentage of body weight loss, with a total of 520 subjects in the experimental group and 407 in the control group [13,15,17-19,21,22]. Regarding the combined effect, for the random-effects model, the MDs evidenced a significant impact in the decreased of body weight of -4.53% (95% CI -5.43 to -3.63) (SMD -3.42 95% CI -5.89 to -0.95) compared to the control group (p < 0.00001); however, there is high heterogeneity between studies (I^2^ = 92%) (Figure 3).

**Figure 4:**
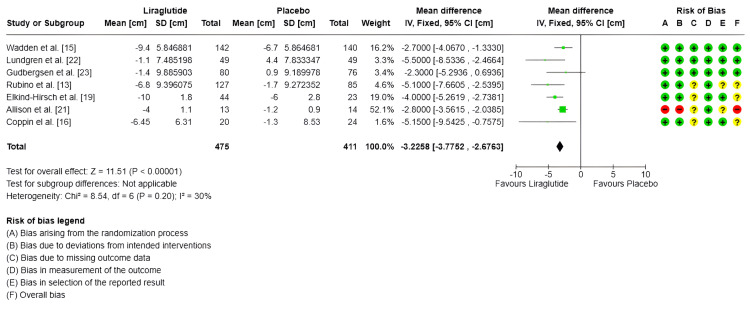
Forest plot and risk of bias: change in waist circumference (cm) IV: Inverse variance; Random: Random-effects model; Std.: Standardized; 95% CI: 95% confidence interval; + Low risk of bias; ? Some concerns; - High risk of bias References: [15,22,23,13,19,21,16]

Waist circumference (cm): Only seven studies measured waist circumference, with a total of 475 subjects in the experimental group and 411 in the control group [13,15,16,19,21-23]. Regarding the combined effect, for the fixed-effects model, the MDs evidenced a significant impact in the decreased of waist circumference of -3.22 cm (95% CI -3.77 to -2.67) (SMD -0.58 95% CI -0.72 to -0.45) compared to the control group (p < 0.00001); (I^2^ = 30%) (Figure 4).

**Figure 5:**
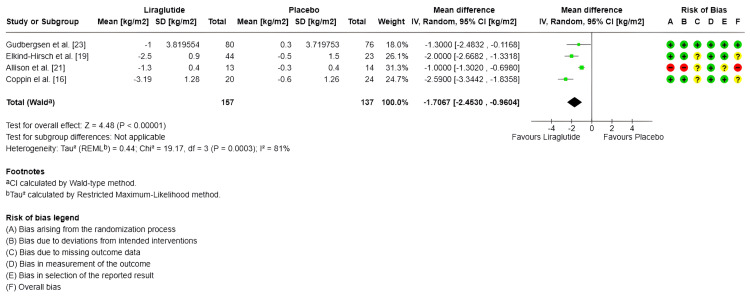
Forest plot and risk of bias: change in body mass index (kg/m2) IV: Inverse variance; Random: Random-effects model; Std.: Standardized; 95% CI: 95% confidence interval; + Low risk of bias; ? Some concerns; - High risk of bias References: [23,19,21,16]

BMI (kg/m^2^): Only four studies measured BMI, with a total of 157 subjects in the experimental group and 137 in the control group [16,19,21,23]. Regarding the combined effect, for the random-effects model, the MDs evidenced a significant impact in the decreased of BMI of -1.71 kg/m^2^ (95% CI -2.45 to -0.96) (SMD -1.55 95% CI -2.47 to -0.63) compared to the control group (p < 0.00001); however, there is high heterogeneity between studies (I^2^ = 81%) (Figure 5).

**Figure 6:**
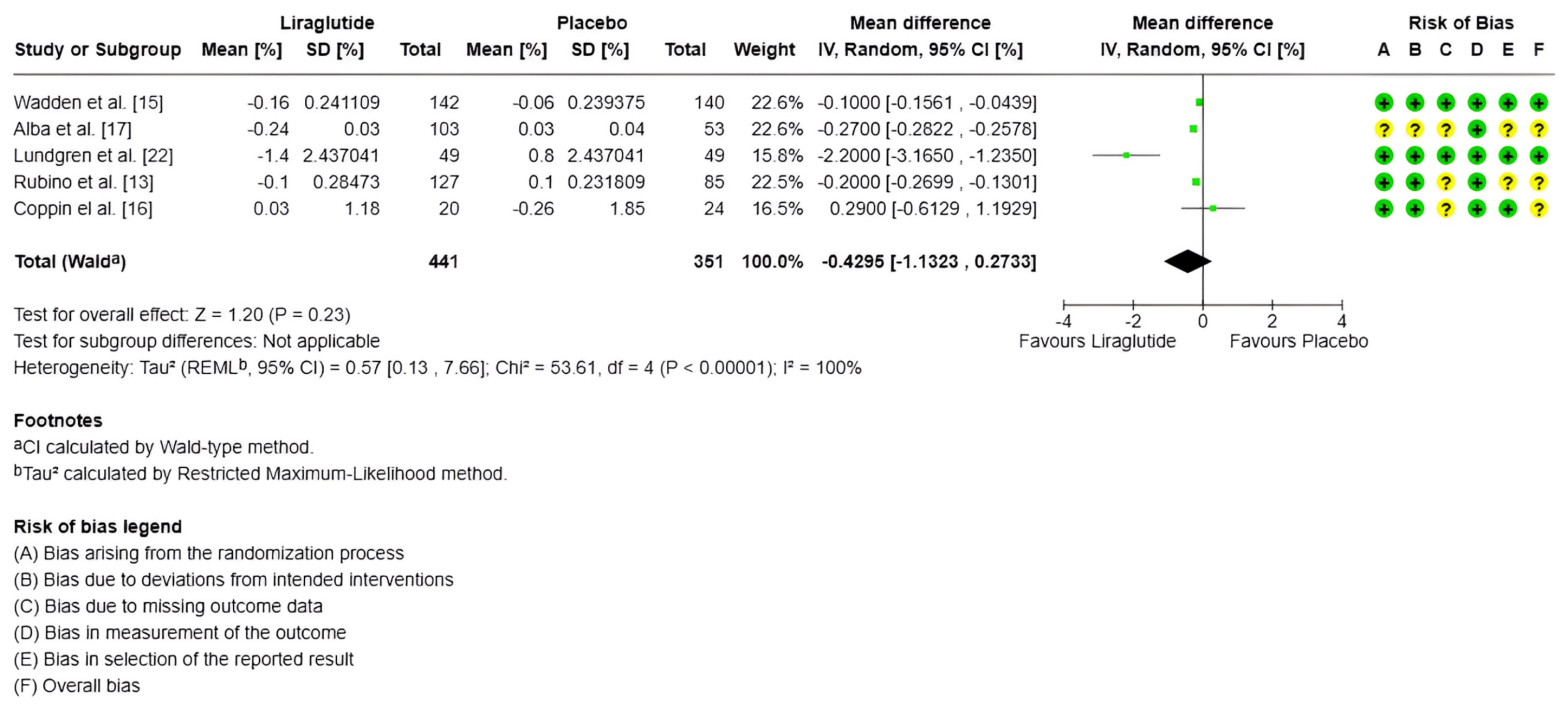
Forest plot and risk of bias: change in HbA1c (glycated hemoglobin) IV: Inverse variance; Random: Random-effects model; Std.: Standardized; 95% CI: 95% confidence interval; + Low risk of bias; ? Some concerns; - High risk of bias References: [15,17,22,13,16]

HbA1c: Only five studies measured HbA1c, with a total of 157 subjects in the experimental group and 137 in the control group [16,19,21,23]. Regarding the combined effect, for the random-effects model, the MDs showed no statistically significant effect in the decrease of HbA1c of -0.43% (95% CI -1.13 to 0.27) (SMD -1.95 95% CI-4.88 to 0.98) compared to the control group (p < 0.00001); however, there is a high heterogeneity between studies (I^2^ = 100%) (Figure 6).

Sensitivity Analysis

It was observed that the main results remained consistent even after adjusting for factors such as the risk of bias and variations in treatment duration. Although adjustments were made, no significant inconsistencies were found in the effects of liraglutide on weight, waist circumference, and BMI. This indicates that the results of the meta-analysis are robust and that the conclusions regarding the efficacy of liraglutide for weight reduction are reliable, despite the variations among the included studies.

Risks of Bias of the Included Studies

**Figure 7:**
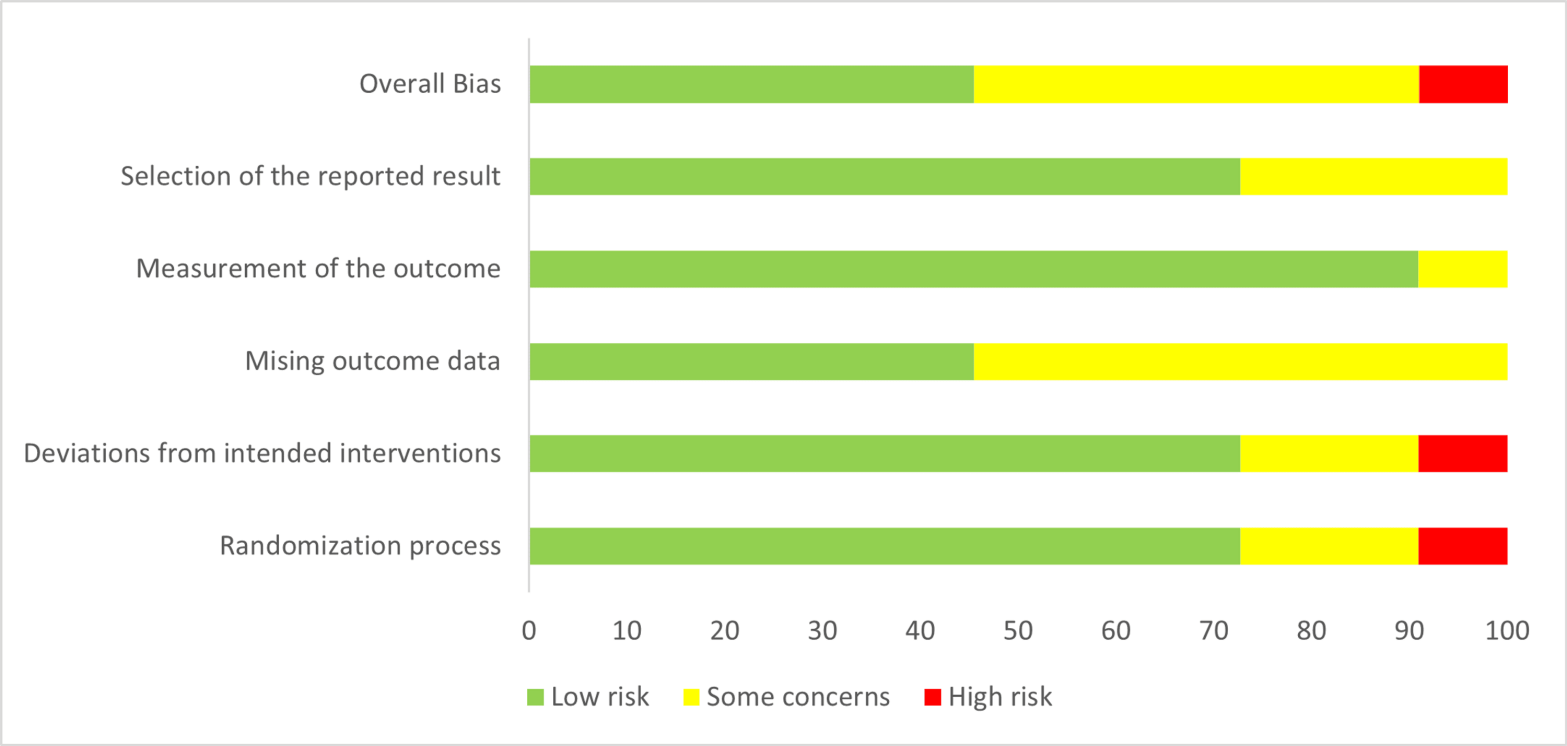
Risk of bias: each factor is presented as percentages overall among the included studies

Figure 7 illustrates the assessment of risk of bias across different domains, based on the Cochrane risk-of-bias tool. While the selection of reported outcomes and measurement of outcomes predominantly present low risk, both show some concerns. Missing outcome data shows a higher proportion of concerns, while deviations from intended interventions and the randomization process show significant concerns, the latter also showing a high-risk component. Overall, although several domains show low risk, concerns and high-risk elements, particularly in randomization and intervention deviations, pose potential threats to the internal validity of the studies.

Publication Bias for Change in Body Weight (Kg)

**Figure 8:**
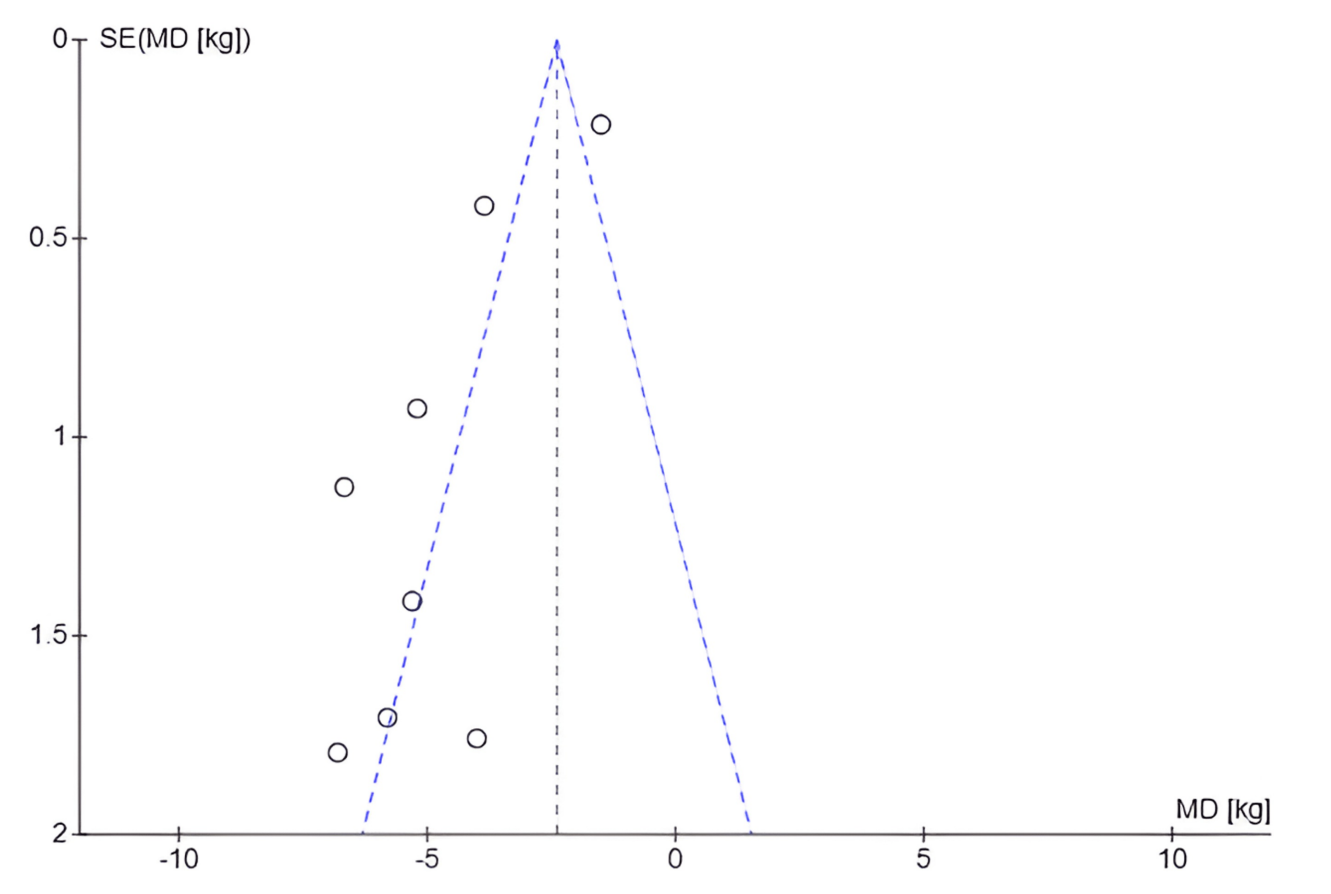
Funnel plot: body weight (kilograms) SE: Standard error; MD: Mean difference

Figure 8 suggests the possibility of publication bias, as studies with small effects appear to be underrepresented, which may indicate that studies with nonsignificant or unfavorable results were not published or included in the analysis. In addition, the dispersion of data points around the central line, rather than a symmetrical funnel-shaped distribution, reinforces this interpretation. This potential asymmetry could affect the validity of the meta-analysis conclusions because the absence of studies with smaller effects on the outcome of interest could lead to an overestimation of the intervention's effect. The observed asymmetry could be due to factors such as publication bias, methodological heterogeneity among studies, or variability in intervention effects.

Publication Bias for Body Weight Loss (%)

**Figure 9:**
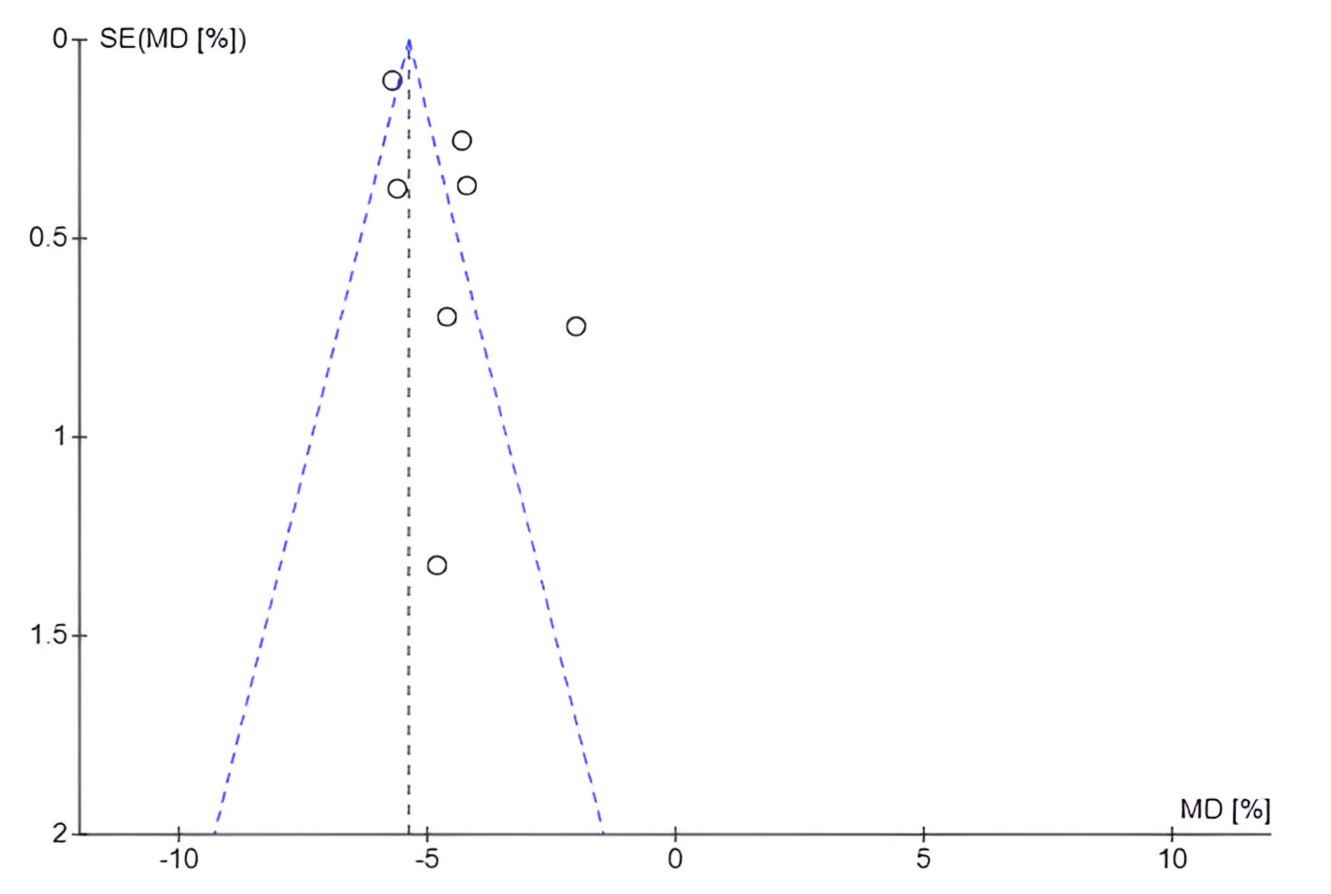
Funnel plot: body weight loss (%) SE: Standard error; MD: Mean difference

Figure 9 shows an asymmetric distribution, suggesting the potential presence of publication bias. This implied that studies with smaller or null effects on weight loss may have been underrepresented, potentially affecting the validity and generalizability of the meta-analysis results.

Publication Bias for Waist Circumference (Cm)

**Figure 10:**
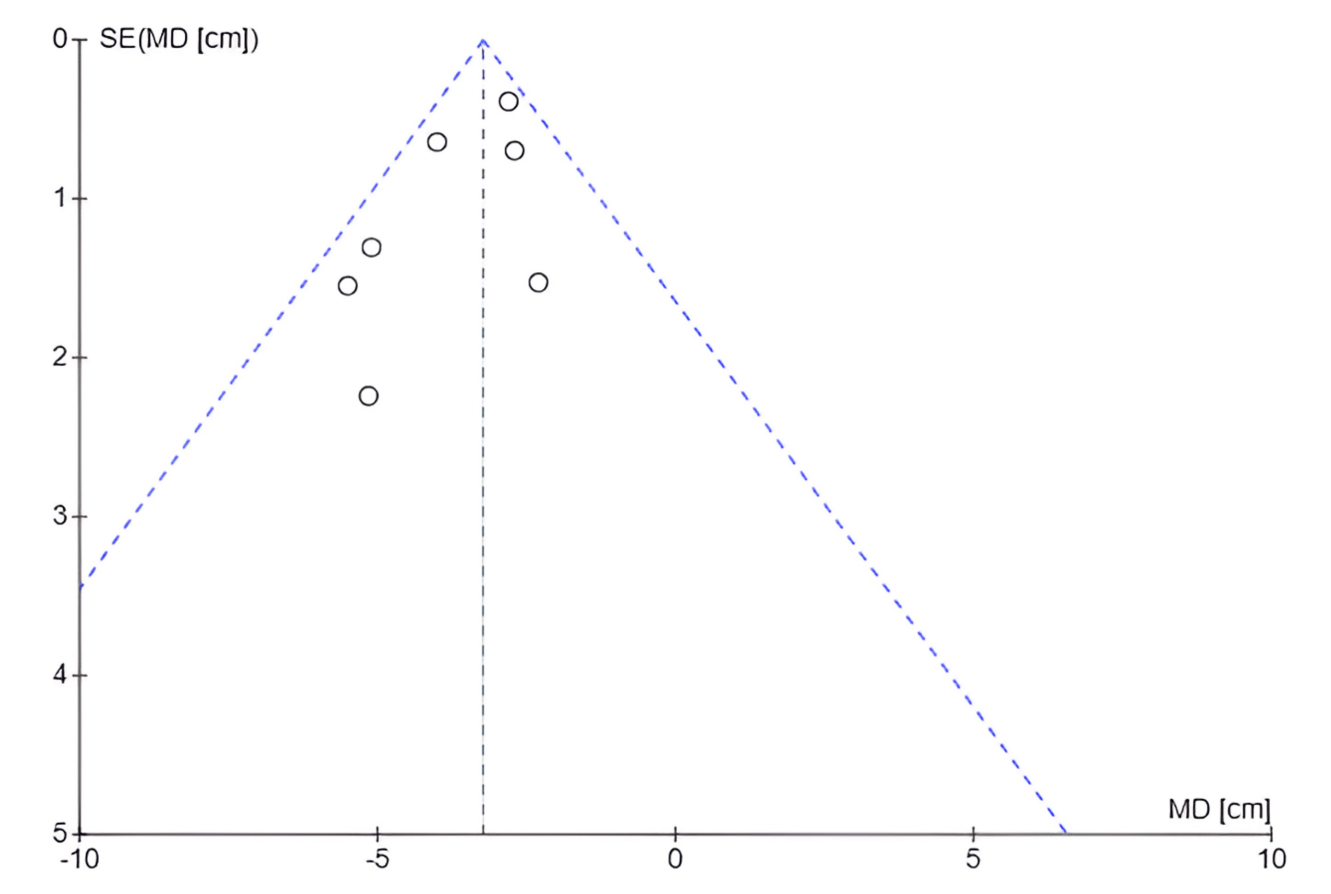
Funnel plot: waist circumference (cm) SE: Standard error; MD: Mean difference

Figure 10 shows a possible asymmetry in the studies included in the meta-analysis. This could indicate the presence of publication bias, heterogeneity of results, or under-representation of smaller studies with positive effects.

Discussion

The results obtained in this review provide compelling evidence that liraglutide was effective in reducing body weight, percentage of weight loss, waist circumference, and BMI. In addition, it contributed to the improvement of certain metabolic parameters such as HbA1c, making it an effective therapeutic option for the management of non-diabetic patients with obesity and overweight.

Evidence that treatment with liraglutide 3.0 mg is consistently associated with significant reductions in body weight and BMI, and is more effective in people without diabetes, comes from multiple studies [24-26], including meta-analyses and clinical trials in different populations (overweight or obese adults, patients with regain or inadequate weight loss after bariatric surgery, and people with hypothalamic obesity).

This study, based on evidence from the past five years, confirms that liraglutide is effective for weight loss in overweight or obese adults without diabetes, with a mean loss of -4.59 kg (95% CI -6.02 to -3.15). Although clinically relevant, this effect was smaller than that observed in a previous meta-analysis of GLP-1 RAs [27], which reported a loss of -7.1 kg (95% CI -9.2 to -5.0), possibly because of the type of agonist, the period of analysis, and methodological differences.

This review shows an overall favourable safety profile. However, the rate of discontinuation due to adverse events is higher in the liraglutide-treated groups. This is in line with previous controlled clinical trials of higher rates of adverse events and discontinuation in this patient population [24,28], where gastrointestinal side effects such as nausea, vomiting, dyspepsia, diarrhea, constipation, and abdominal pain were the most common adverse events. In addition, a higher rate of adverse events and discontinuations was observed in patients treated with liraglutide than in those treated with placebo, although no serious adverse events were reported.

The results of the studies analyzed indicated a higher incidence of adverse effects in the liraglutide-treated groups compared with the placebo control groups. In the clinical trials by Rubino et al. (96.1% vs. 95.3%) [13], Wadden et al. (95.8% vs. 88.6%) [15], Alba et al. (80.7% vs. 71.7%) [17], Saxena et al (96.87% vs. 71.42%) [20], and Allison et al. (89.5% vs. 64.7%) [21], an increased incidence of adverse events was observed in the experimental group.

The most common adverse events were gastrointestinal, mainly nausea, vomiting, and diarrhoea, which are usually transient. No deaths were reported, and serious adverse events were rare. These results suggest that the use of liraglutide is associated with an increased likelihood of adverse effects compared with placebo, which should be considered in the risk-benefit assessment of treatment.

Combating obesity is critical because it increases all-cause mortality and is associated with comorbidities such as heart failure, thromboembolic disease, obstructive sleep apnea, pulmonary hypertension, diabetes, cancer, gallbladder disease, pain, etc. [29-31].

Limitations

The inclusion of only 11 trials may reduce the generalizability of the findings, especially because of the small sample sizes of the individual trials. The included studies are heterogeneous in terms of design, intervention, population studied, length of follow-up, and outcome measures. This may make direct comparisons difficult and affect the validity of the results. In addition, the included studies do not stratify outcomes for overweight and obesity separately, and the funnel plot indicates a potential publication bias.

Finally, we believe that most of the trials were conducted in developed countries, thus limiting their applicability at the local level in less developed countries, where resources and health conditions may differ considerably.

## Conclusions

Administration of liraglutide at a daily dose of 3.0 mg shows significant efficacy in inducing weight loss in overweight or obese adults without a diagnosis of type 2 diabetes mellitus, compared with placebo. Results show consistent improvements in reductions in body weight, waist circumference, and BMI, supporting its utility as an additional pharmacologic intervention in the comprehensive approach to obesity and overweight in this population.
