# Correction: Efficacy of Liraglutide for Weight Loss in Overweight and Obese Non-diabetic Adults: A Systematic Review and Meta-Analysis of Randomized Controlled Trials

**DOI:** 10.7759/cureus.c277

**Published:** 2025-08-27

**Authors:** Néstor Israel Quinapanta Castro, Mishell Almeida, Andres F Orbea, Domenica N Andrade, Jonathan Flores Carrera, Francisco Yepez Vargas, Mariela L Carrasco

**Affiliations:** 1 Department of Research, Regional Autonomous University of the Andes (UNIANDES), Ambato, ECU; 2 Department of Medicine, Central University of Ecuador, Quito, ECU; 3 Department of Medicine, Regional Autonomous University of the Andes (UNIANDES), Ambato, ECU; 4 Department of Occupational Health, Pontifical Catholic University of Ecuador, Quito, ECU; 5 Department of Emergency Medicine, Ambato General Hospital (IESS), Ambato, ECU

This article has been corrected as follows: the second paragraph of the Abstract has been corrected to read ‘Risk of bias was assessed using the Cochrane Risk of Bias 2.0 (RoB 2.0) tool for randomized trials’. The original version incorrectly included ‘Cochrane Risk of Bias tool for observational studies’ instead of ‘Cochrane Risk of Bias tool for randomized trials’.

